# A Comparison of Methods to Measure the Coupling Coefficient of Electromagnetic Vibration Energy Harvesters

**DOI:** 10.3390/mi10120826

**Published:** 2019-11-28

**Authors:** Mario Mösch, Gerhard Fischerauer

**Affiliations:** Chair of Measurement and Control Systems, Center of Energy Technology (ZET), Universität Bayreuth, Universitätsstraße 30, D-95447 Bayreuth, Germany; mrt@uni-bayreuth.de

**Keywords:** vibration energy harvesting, electromagnetic, coupling, measurement, simulation, microgenerator

## Abstract

Vibration energy harvesters transform environmental vibration energy into usable electrical energy. The transformation is only possible because of a coupling between the mechanical part of the energy harvester and the electric circuit. This paper compares several measurement methods to determine the electromagnetic coupling coefficient. These methods are applied to various implementations of an energy harvester and the results are compared with one another and with simulation data by analyzing the magnetic flux. The average deviation between the measurement methods and the simulation data in our study was 5%. This good agreement validates the methods. Based on this, we recommend determination of the coupling coefficient and the optimum load resistance for maximum power harvesting on the basis of simulations and the open circuit method, because this procedure leads to the shortest measurement times.

## 1. Introduction

The deployment of many wireless sensor networks only makes sense commercially and environmentally if the sensors can be powered by energy harvesting (i.e., by converting energy freely available in the environment to usable electrical energy) [1]. The energy sources can be vibration, light, temperature gradients, airflow, or electromagnetic waves [2,3]. Vibration energy can be converted by electromagnetic, piezoelectric, or electrostatic principles [4]. Electromagnetic conversion is achieved by the relative movement of a permanent magnet and a coil, in which a voltage is induced.

Vibration harvesters are designed for a specific environment. The energy output is maximized when the resonance frequency matches the vibration frequency. In time-variant environments, the average energy output can be increased by adapting the resonance frequency of a narrowband harvester to the instantaneous vibration frequency or by using a broadband harvester [5,6,7]. An example of the latter case are nonlinear transducers [8,9].

An essential aspect of vibration energy harvesters is the coupling between the mechanical and electrical part of harvester. In piezoelectric harvesters, this coupling is described by the piezoelectric coefficient [10,11], while in electromagnetic harvesters it is described by the electromagnetic coupling coefficient [12,13].

Several authors have dealt with the simulation or measurement of the latter. Glynne-Jones et al. [14] experimentally investigated the optimum load resistance leading to the maximum generated power. They also derive the magnetic flux density from the induction voltage, but did not deal with the coupling. Stephen [12] derived the coupling coefficient and its influence on the optimum load resistance in detail, but did not provide experimental validation. O’Donnell et al. [13] investigated the coupling by finite element analysis (FEA), but again no measurements were performed to corroborate the simulation results.

Cheng et al. [15] derived a lumped element equivalent circuit of electromagnetic energy harvesters, built a prototype, and measured its parameters. The coupling was determined by applying a current to the pickup coil and measuring the resulting magnetic force; the coupling coefficient then is the slope of the linear fit function between the force and the current. The damping coefficient was measured by an open circuit resonance curve, as well as by the impulse response. The optimum load resistance was estimated by applying different load resistances and measuring the load voltage. This paper is very detailed, but lacks a comparison between simulated and measured coupling coefficients.

Spreemann et al. [16] demonstrated an interesting numerical optimization approach for the parameters of electromagnetic harvesters, such as the resting position of the mass and geometrical magnet and coil parameters. They calculated the magnetic field analytically and reported the normalized gradient of the magnetic flux density. The optimum load resistance of a prototype was measured by applying different load resistances and searching for maximum power. A concrete coupling coefficient value was neither given by calculation nor by measurement.

Mann and Owens [17] investigated a nonlinear electromagnetic energy harvester with a bistable potential well. They reported a measured value of the coupling, but did not clearly state how the value was measured. Overall, the physical dimension of the parameter appears to be wrong. Cepnik et al. [18] described the time-optimized simulation of the electromagnetic coupling by analytical calculation, but did not report simulated or measured values. Szarka et al. [19] examined power conditioning systems for electromagnetic transducers and stated the numerical value of the electromagnetic coupling coefficient of a prototype, but did not reveal how the value was obtained.

It is obvious that the coupling coefficient in electromagnetic energy harvesters has not received sufficient attention up to now. Many values cited in the literature cannot be considered reliable and can hardly be compared to one another for lack of detail. This paper aims at shedding some light on the issue. It deals with the simulation and measurement of the electromagnetic coupling coefficient. Section 2 explains the electromagnetic coupling theory and how the coupling coefficient can be extracted from simulation and measurement data. Section 3 is devoted to the simulation (by FEA), realization, and experimental characterization of a prototype harvester. The results of the measurement methods are discussed in Section 4. Finally, Section 5 gives a summary.

## 2. Electromagnetic Coupling

### 2.1. Theory

**Figure 1:**
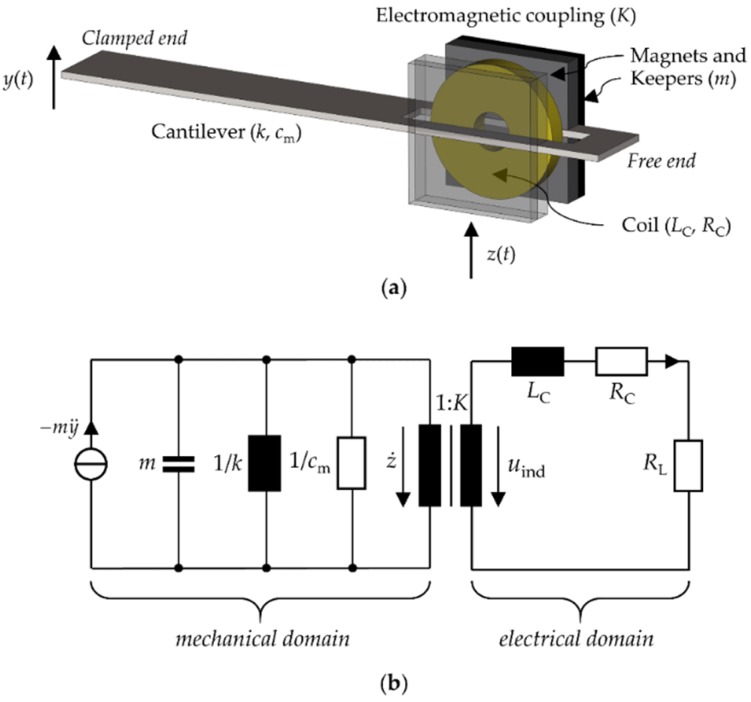
Clamped cantilever as an electromagnetic vibration harvester. (**a**) Mechanical model. The load resistance *R*_L_ is connected to the coil and is not indicated. (**b**) Equivalent circuit [21,22].

Electromagnetic vibration energy harvesters mainly consist of permanent magnets and induction coils. When exposed to vibration, the permanent magnets move relative to the coils, in which a voltage is induced by Faraday’s law. The voltage can be used to power a load. The mechanical part of the harvester is a distributed system and must be described by partial differential equations. In many instances, however, it suffices to track the movement of a representative element or reference point on the harvester, which can be described by ordinary differential equations. In this sense, a light cantilever beam with heavy permanent magnets and iron keepers near its free end can be modeled by an equivalent spring–mass–damper system, with the mass of the magnets and the keepers as effective mass m, an effective spring constant k, and a mechanical damping coefficient $c_{m}$ (Figure 1a). The mass and the spring constant define the natural resonance frequency $\omega_{n}=\sqrt{k/m}$. The electrical part consists of the induction coil with the induction voltage $u_{\mathrm{ind}}$ as the voltage source, $L_{C}$ and $R_{C}$ as coil inductance and resistance, respectively, and $R_{L}$ as load resistance (Figure 1b).

The equivalent lumped element model is excited by a base acceleration $a(t)=-\ddot{y}(t)$ with the base position y. With the relative displacement z(t)=x(t)−y(t) (the absolute displacement x(t) may be taken to coincide with the center of mass of the permanent magnet in the case of the tip-loaded cantilever beam) and the coupling coefficient K, the system is described by the following coupled differential equations [15,18]

(1) $$m\ddot{z}(t)+c_{m}\dot{z}(t)+kz(t)+Ki(t)=-m\ddot{y}(t),$$

(2) $$u_{\mathrm{ind}}(t)=K\dot{z}(t)=(R_{C}+R_{L})i(t)+L_{C}\dot{i}(t).$$

Equation (1), with K=0, is the differential equation of an unloaded mechanical oscillator [20]. The extra term is due to the back action of the induction current in the linear approximation [16].

The left part of Equation (2) is derived from Faraday’s law by

(3) $$u_{\mathrm{ind}}(t)\approx -N\frac{d\varphi }{dt}=-N\frac{d\varphi }{dz}\dot{z}(t)=:K\dot{z}(t)$$

with N the number of coil turns and φ the magnetic flux through a single coil turn [18]. K depends on the coil geometry and the magnetic circuit details. For example, for a given coil volume, K can be increased by using thinner wires and more coil turns.

Neglecting the coil inductance $L_{C}$ in Equation (2) at the commonly low frequencies [13], solving the current i(t) and inserting the result into Equation (1) shows that the back action of the induction current on the mechanical part of the harvester is equivalent to an additional electrical damping coefficient $c_{e}$:

(4) $$c_{e}=\frac{K^{2}}{R_{C}+R_{L}}.$$

Here, $c_{e}$ depends on both the harvester and the load resistance $R_{L}$. With small $R_{L}$, the electrical damping coefficient $c_{e}$, and consequently the total damping coefficient $c=c_{e}+c_{m}$, are high. This reduces the displacement z(t), the velocity $\dot{z}(t)$, the induction voltage $u_{\mathrm{ind}}$, and the load power $P_{L}$. With high $R_{L}$, $c_{e}$ is small, and therefore z(t) and $\dot{z}(t)$ are high, but the load power is again small because the load current is small.

Stephen [12] showed that care must be exercised when looking for the optimum load resistance $R_{L,\mathrm{opt}}$ providing the highest load power. The maximum power transfer theorem would lead one to set $R_{L,\mathrm{opt}}=R_{C}$, but this is not true here because of the back action of the load current. It rather follows from $dP_{L}/dR_{L}=0$ that [12]

(5) $$R_{L,\mathrm{opt}}=R_{C}+\frac{K^{2}}{c_{m}}.$$

### 2.2. Four Methods of Measuring the Electromagnetic Coupling Coefficient

**Figure 2:**
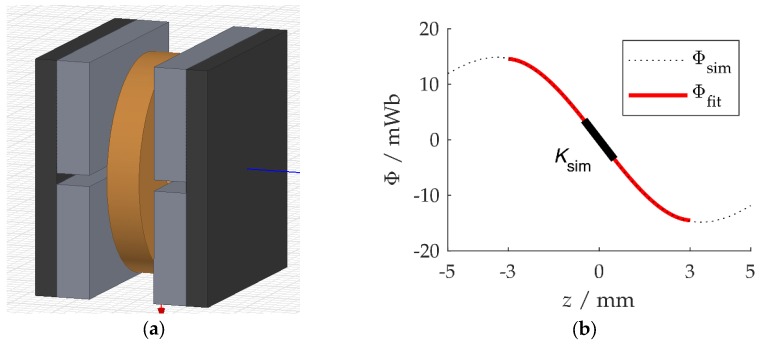
(**a**) Model of a magnetic circuit with coil (copper), magnets (grey), and iron (dark). (**b**) FE simulation result of the magnetic flux with Ansys Electromagnetics.

The above theoretical considerations lead to several methods of extracting the coupling coefficient from simulation or measurement data. First, K can be found by an electromagnetic FEA. Modeling the magnetic circuit and the induction coil shown in Figure 2a in a FE program (e.g. Ansys Electromagnetics) and making the parts move relative to each other yields the total magnetic flux Φ(z)=Nφ(z). Figure 2b shows an exemplary curve with a third-order polynomial fitting function. The linear gradient of the curve at z=0 is the simulated coupling coefficient $K_{\mathrm{sim}}$ used for comparison in this work. $K_{\mathrm{sim}}$ is approximately valid for small oscillation amplitudes. The geometry and the simulation details behind Figure 2 are respectively described in Section 3.1 and Section 3.2.

**Figure 3:**
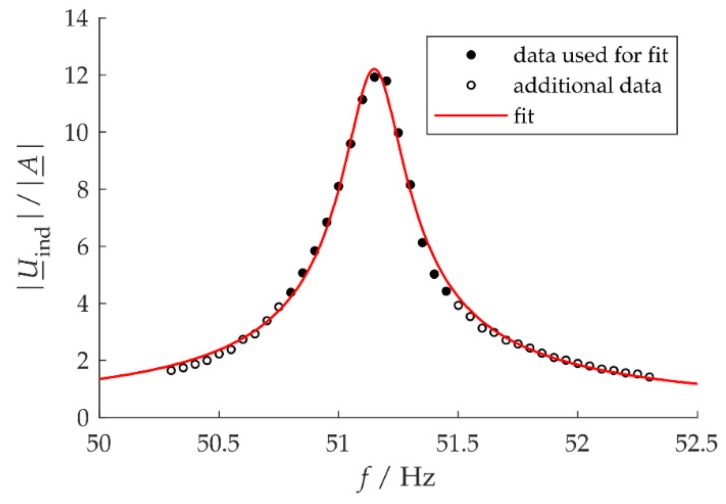
Measured resonance curve of the induced voltage in an electromagnetic energy harvester and best fit by a curve of the type given in Equation (6) for setup 1L from Section 3.3 below.

To extract coupling coefficient values from measurements, the base of an energy harvester is excited harmonically with constant acceleration amplitude $\hat{a}$ and varying frequencies. With $R_{L}\to \infty$, the measured open circuit voltage is the induction voltage. The transfer function, defined as the ratio of the induction voltage phasor ${\underline{U}}_{\mathrm{ind}}$ to the acceleration phasor $\underline{A}$, is derived from Equation (1) (underlined symbols are understood to represent complex-valued quantities.) Its magnitude is

(6) $$|\frac{{\underline{U}}_{\mathrm{ind}}}{\underline{A}}|=K\frac{\omega /\omega_{n}{}^{2}}{\sqrt{(1-{\left(\omega /\omega_{n}\right)}^{2})+{\left(2\zeta_{m}\omega /\omega_{n}\right)}^{2}}}.$$

with the mechanical damping factor $\zeta_{m}=c_{m}/(2\sqrt{km})$. Fitting measured data with functions of this type and choosing the parameter $\zeta_{m}$ for optimum agreement between measured data and fit curve allows one to identify the coupling coefficient K (Figure 3). The corresponding estimate of K will be called $K_{\mathrm{oc}}$ in the following.

The resonance curve of the absolute position x(t) in the case of a short circuit ($R_{L}=0$) yields the transfer function magnitude.

(7) $$|\frac{\underline{X}}{\underline{Y}}|=\sqrt{\frac{1+{\left(2(\zeta_{m}+\zeta_{e,\mathrm{sc}})\omega /\omega_{n}\right)}^{2}}{(1-{\left(\omega /\omega_{n}\right)}^{2})+{\left(2(\zeta_{m}+\zeta_{e,\mathrm{sc}})\omega /\omega_{n}\right)}^{2}}}.$$

Here, $\underline{X}$ and $\underline{Y}$ are the phasors of x(t) and y(t). Fitting such a curve to measured data respectively yields the short-circuit damping factor $\zeta_{e,\mathrm{sc}}$ and the electrical damping coefficient $c_{e,\mathrm{sc}}$. From this, an estimate $K_{\mathrm{sc}}$ of the coupling coefficient is obtained from Equation (4) by

(8) $$K_{\mathrm{sc}}=\sqrt{R_{C}\cdot c_{e,\mathrm{sc}}}=\sqrt{R_{C}\cdot (c(R_{L}=0)-c_{m})}.$$

This method requires knowledge of the mechanical damping coefficient $\zeta_{m}$, and therefore serves as additional validation of the open circuit method.

A third way of estimating the coupling coefficient is by evaluating the measured optimum load resistance $R_{L,\mathrm{opt}}$. The load voltage spectrum near resonance is measured with different load resistances, and the resistance resulting in the highest power at resonance is $R_{L,\mathrm{opt}}$ [14,15,16]. An estimate of the coupling coefficient then follows from Equation (5):

(9) $$K_{R}=\sqrt{(R_{L,\mathrm{opt}}-R_{C})\cdot c_{m}}.$$

The procedure is time consuming because one has to determine the peaks of resonance curves for various load resistances. It does not suffice to just measure the output power at a fixed frequency (e.g., the open circuit resonance frequency) because the resonance frequency varies with the damping [20].

The fourth measurement method for the coupling coefficient is based on a linear variation of the current i through the coil [15]. The resulting magnetic force $F_{\mathrm{mag}}$ on the permanent magnets is measured by the cantilever beam deflection x via the effective spring constant k. One obtains the following estimate of the coupling coefficient $K_{i}$:

(10) $$K_{i}=\frac{F_{\mathrm{mag}}}{i}=\frac{kx}{i}.$$

This can be exploited fully automatically with a slowly rising and falling current. The slow change reduces measurement errors because the mass is slowly swinging. The spring constant can be taken from $k=m\omega_{n}{}^{2}$, which can be measured with a force testing system or can be extracted from simulations.

When the mechanical damping coefficient and the coupling coefficient are known, the optimum load resistance can be predicted by Equation (5). A comparison between the measured load resistance $R_{L,\mathrm{opt}}$ and the calculated one, $R_{L,\mathrm{opt},c}$, provides a measure of goodness of the measurement and the simulation procedures.

## 3. Experimental and Simulative Validation

### 3.1. Energy Harvester Implementation

**Figure 4:**
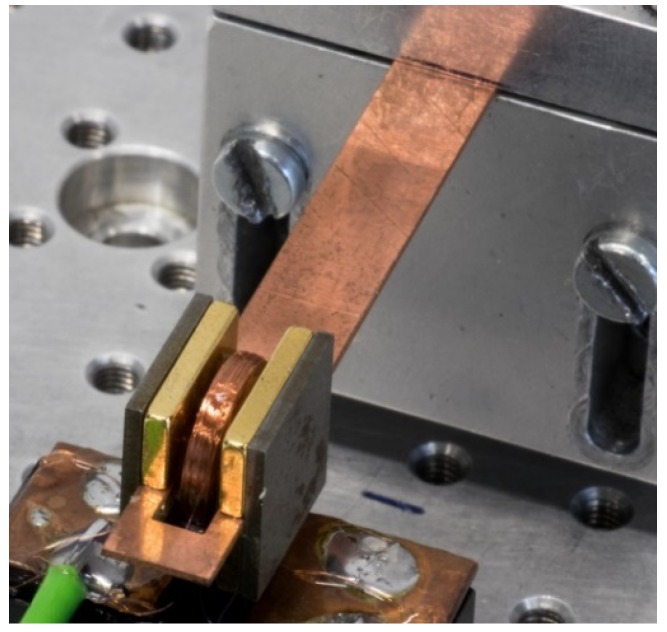
Photograph of the cantilever energy harvester used for experimental tests. The reader sees the clamped copper beam, two magnets with golden coating, the iron legs of the magnetic circuit, and the copper coil in the gap between the magnets (connected to the base).

To compare the respective characteristics of the various measurement methods for the coupling coefficient, we applied the methods to a typical vibration energy harvester type. This type consists of a cantilever made from copper, of four NdFeB magnets, and of two iron legs to guide the magnetic flux (Figure 4). The seismic mass at the end of the cantilever weighed m=4.07g. The magnet grade was N50, with a remnant flux density of $B_{r}=1.43T$ and a coercive field strength of $H_{c}=955\mathrm{kA}/m$. The respective dimensions of the magnets and the iron legs were 10 mm × 5 mm × 1 mm and 10.5 mm × 10 mm × 1 mm. The copper beam was 0.5 mm thick and comprised a rectangular slot 14 mm long and 3 mm wide, in which an induction coil could freely move up and down. The shortest distances of the cantilever end and the clamping point to the slot were 3 mm and ℓ, respectively. Hence, the full cantilever length amounted to ℓ+14mm+3mm.

Various implementations of the harvester type described were used in the experiments. The implementations differed in the free clamping lengths ℓ and the coils. Three different coils were tested. They all had identical outer dimensions (inner and outer diameters $D_{i}=2.9\mathrm{mm}$ and $D_{o}=9.7\mathrm{mm}$, respectively, and height h=1.49mm), but different wire diameters. Enameled copper wires with diameters $D_{w}$ between 30 and 50 µm were used, and the value of $D_{w}$ determined the number of coil turns N and the coil resistance $R_{C}$. Further values are listed in Table 1.

The absolute uncertainties of the magnet and cantilever geometries were about 0.1 mm, the outer coil dimension uncertainty amounted to 0.2 mm according to the producer, and the remaining coil dimension uncertainties were negligible. The residual flux density uncertainty of the magnets was 30 mT according to the data sheet. Finally, the relative uncertainties of the mass and of the coil resistance were 1%, caused by the scales and multimeters used.

### 3.2. Finite Element Simulation

The magnetic circuit with the four magnets and the coil was modeled by the FE program Ansys Electromagnetics (Figure 2; dimensions and magnet data as in Section 3.1). The FE mesh comprised over 10,000 tetrahedron elements. The magnetic flux Φ through the coils vanishes in the quiescent (equilibrium) state z=0, where z denotes the relative displacement of the magnets and the coils, because the magnets generate a quadrupole field. In the simulation, the dependence of the magnetic flux on the displacement, Φ(z) was easily obtained by varying z. The effects of dimensional and magnet parameter deviations were studied in the same manner.

### 3.3. Measurement Details

On the one hand, we characterized harvesters with identical clamping lengths ℓ, different coils, and an identical harmonic acceleration amplitude of $\hat{a}=0.75{m/s}^{2}$. On the other hand, we tested harvesters with identical coils, different clamping lengths, and an identical acceleration amplitude of $\hat{a}=1{m/s}^{2}$.

The energy harvesting test bench consisted of a waveform generator connected to a permanent magnet shaker (B&K LDS V406, Nærum, Denmark), an accelerometer (B&K 4534-B-001) for measuring the vibration strength, and a voltage measurement card (NI PIXe-6341, Austin, TX, USA). The measurement card captured the load or open circuit voltages and the voltage from the acceleration sensor. The absolute movement was tracked with a triangulation laser sensor (Micro-Epsilon optoNCDT 2300, Ortenburg, Germany). The load resistance was set by a resistance decade box. The whole bench was controlled by a PC, so the user could vary the frequency and the acceleration amplitude. The center frequency and span of the measurement window in the frequency domain were respectively adjusted to the resonance frequency and a value larger than the resonance-curve bandwidth of the harvester to be characterized. The step width was set to 50 mHz. The voltage and movement resonance curves of all setups are contained in Supplementary Materials.

In the experiments, the actual acceleration amplitudes deviated from the preset values. If ignored, this would introduce errors, as both the load voltage and the cantilever tip displacement are proportional to the acceleration. Therefore, we converted the measured voltages and displacements to the values that would have resulted at the preset reference acceleration. The relative uncertainties of the measured voltages, displacements, and accelerations did not exceed 1%, and were neglected with respect to model and dimensional errors.

The load power was calculated by $P_{L}=U^{2}/R_{L}$, with U being the load voltage. The optimum load resistance $R_{L,\mathrm{opt}}$ is the resistance maximizing the power. $R_{L}$ was changed in steps by way of the resistance decade box.

### 3.4. Propagation of Uncertainty

The electromagnetic coupling coefficient and the optimum load resistance are deduced from other parameters with uncertainties as discussed above. We assume that these parameter uncertainties result from independent and normally distributed random errors. We may then write as

(11) $$u_{K_{\mathrm{sim}}}=\sqrt{\sum_{i}{\left(S_{x_{i}}^{K_{\mathrm{sim}}}u_{x_{i}}\right)}^{2}}=K_{\mathrm{sim}}{\tilde{u}}_{K_{\mathrm{sim}}}$$

where $u_{K_{\mathrm{sim}}}$ and ${\tilde{u}}_{K_{\mathrm{sim}}}$ are the absolute and relative uncertainties of the simulated coupling coefficient, respectively, $x_{i}$ is the uncertain input parameters, and $S_{x_{i}}^{K_{\mathrm{sim}}}=\partial K_{\mathrm{sim}}/\partial x_{i}$ is the sensitivity of $K_{\mathrm{sim}}$ with respect to $x_{i}$ [23]. The remnant flux density $B_{r}$, the gap width $w_{g}$, and the outer coil dimension $D_{o}$ are likely to be the most uncertain influence quantities. We, therefore, use Equation (11) with these three parameters only ($x_{1}=B_{r}$ etc.) and neglect the remaining parameters:

(12) $$u_{K_{\mathrm{sim}}}=\sqrt{{\left(S_{B_{r}}^{K_{\mathrm{sim}}}u_{B_{r}}\right)}^{2}+{\left(S_{w_{g}}^{K_{\mathrm{sim}}}u_{w_{g}}\right)}^{2}+{\left(S_{D_{o}}^{K_{\mathrm{sim}}}u_{D_{o}}\right)}^{2}}$$

The numbers for setups 1 to 3 are $u_{B_{r}}=0.03T$, $u_{w_{g}}=0.1\mathrm{mm}$, $u_{D_{o}}=0.2\mathrm{mm}$, $S_{B_{r}}^{K_{\mathrm{sim}}}=1.62\mathrm{Wb}/(m\cdot T)$, $S_{w_{g}}^{K_{\mathrm{sim}}}=2.13\mathrm{Wb}/(m\cdot \mathrm{mm})$, and $S_{D_{o}}^{K_{\mathrm{sim}}}=0.36\mathrm{Wb}/(m\cdot \mathrm{mm})$. This results in ${\tilde{u}}_{K_{\mathrm{sim}}}\approx 3.3\%$.

The uncertainty of the predicted optimum load resistance $R_{L,\mathrm{opt},c}$ is calculated with the simulated coupling coefficient $K_{\mathrm{sim}}$. The uncertainties of $K_{\mathrm{oc}}$ and the open- and closed-circuit damping factor values $\zeta_{m}$ and $\zeta_{0}$ follow from the fit of Equations (6) and (7). The uncertainties for $R_{L,\mathrm{opt},c}$, $K_{\mathrm{sc}}$, $K_{R}$, and $K_{i}$ are derived from Equations. (5), (8), (9), and (10), respectively, using Equation (11) and $c=4\pi f_{n}\zeta m$:

(13) $$u_{R_{L,\mathrm{opt},c}}=\sqrt{u_{R_{C}}^{2}+{\left(\frac{K_{\mathrm{sim}}^{2}}{4\pi mf_{n}\zeta_{m}}\right)}^{2}(4{\tilde{u}}_{K_{\mathrm{sim}}}^{2}+{\tilde{u}}_{\zeta_{m}}^{2}+{\tilde{u}}_{m}^{2}+{\tilde{u}}_{f_{n}}^{2})},$$

(14) $${\tilde{u}}_{K_{\mathrm{sc}}}=\frac{1}{2}\sqrt{{\tilde{u}}_{R_{C}}^{2}+{\tilde{u}}_{m}^{2}+{\tilde{u}}_{f_{n}}^{2}+\frac{u_{\zeta_{0}}^{2}+u_{\zeta_{m}}^{2}}{{\left(\zeta_{0}-\zeta_{m}\right)}^{2}}},$$

(15) $${\tilde{u}}_{K_{R}}=\frac{1}{2}\sqrt{{\tilde{u}}_{\zeta_{m}}^{2}+{\tilde{u}}_{m}^{2}+{\tilde{u}}_{f_{n}}^{2}+\frac{u_{R_{L,\mathrm{opt}}}^{2}+u_{R_{C}}^{2}}{{\left(R_{L,\mathrm{opt}}-R_{C}\right)}^{2}}},\mathrm{and}$$

(16) $${\tilde{u}}_{K_{i}}=\frac{1}{2}\sqrt{{\tilde{u}}_{R_{C}}^{2}+{\tilde{u}}_{m}^{2}+4{\tilde{u}}_{f_{n}}^{2}}.$$

## 4. Results and Discussion

### 4.1. Damping Influence and Optimum Load

**Figure 5:**
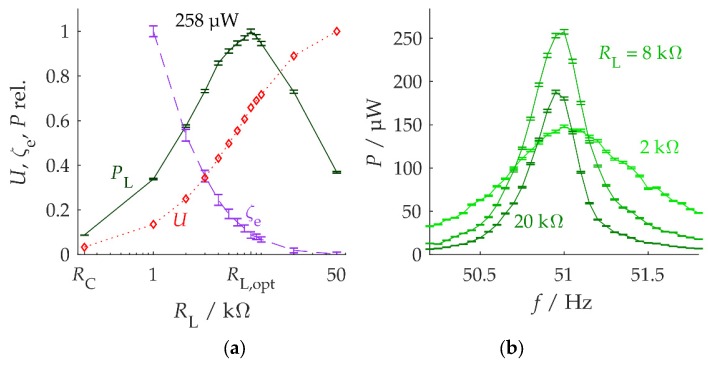
Measured load-resistance influence in harvester setup 1L. (**a**) Voltage amplitude, electrical damping factor, and maximum load power as functions of the load resistance. All quantities have been normalized for unity. The voltage uncertainties are negligible, and therefore have not been indicated. (**b**) Frequency dependence of the power delivered to the load for different load resistances. The optimum load resistance for harvester excitement at its resonance frequency of about 51 Hz is 8 kΩ.

Figure 5 shows the influence of the load resistance in a harvester setup 1L at a vibration amplitude of $\hat{a}=0.75{m/s}^{2}$. The voltage resonance curves for all measured load resistances are contained in Supplementary Materials. The load voltage amplitude U, the electrical damping factor $\zeta_{e}$, and the power $P_{L}$ delivered to the load at the resonance frequency all depend on the load resistance $R_{L}$ (Figure 5a). The expectations from Section 2.1 are met: for a low load resistance (e.g., $R_{L}=R_{C}$), the damping factor $\zeta_{e}$ is large and the displacement is small, thus limiting the load power. A high load resistance leads to a reduced damping factor, but this is not enough to offset the current reduction, which again limits the load power. The optimum load resistance $R_{L,\mathrm{opt}}$ lies between the limiting cases of small and high values. Surprisingly, $R_{L,\mathrm{opt}}$ is an order of magnitude higher than the coil resistance $R_{C}$ (Table 2).

The frequency dependence of the load power is in agreement with these observations (Figure 5b). Any load different from $R_{L,\mathrm{opt}}$ leads to a lower load power at resonance, $P_{\max}$. The much higher damping associated with small $R_{L}$ can be used for frequency adaptivity: at vibration frequencies near (but not equal to) the harvester resonance frequency, the flat resonance curve for small $R_{L}$ effectively leads to more load power than the narrowband resonance curve for $R_{L,\mathrm{opt}}$.

The maximum load powers of our harvesters with the long free clamping lengths (setups 1L, 2, and 3) amounted to between 223 and 257 µW at $\hat{a}=0.75{m/s}^{2}$. In Section 2.1, it was mentioned that a higher number of coil turns N increases the coupling coefficient K. From Equation (3), we may write $K=C_{K}N$ with a constant $C_{K}$ independent of N. However, the load power does not depend on N when the coil dimensions remain unchanged, the load resistance is optimum, and the system is operated at resonance. To understand this, first note that the coil resistance is approximately

(17) $$R_{C}=C_{R}N^{2}$$

with a constant $C_{R}$ independent of N (cf. derivation in Appendix A). The time-averaged load power then becomes [12]

(18) $$P=\frac{m^{2}{\hat{a}}^{2}}{8c_{m}}{\left(1+c_{m}\frac{R_{C}}{K^{2}}\right)}^{-1}=\frac{m^{2}{\hat{a}}^{2}}{8c_{m}}{\left(1+c_{m}\frac{C_{R}N^{2}}{C_{K}^{2}N^{2}}\right)}^{-1}\neq f(N)$$

Up until non-idealities and measurement errors, our measurement results demonstrate this independence of the power on the number of coil turns (Table 2). In contrast, the free clamping length ℓ clearly has an influence.

### 4.2. Measuring $K_{i}$

**Figure 6:**
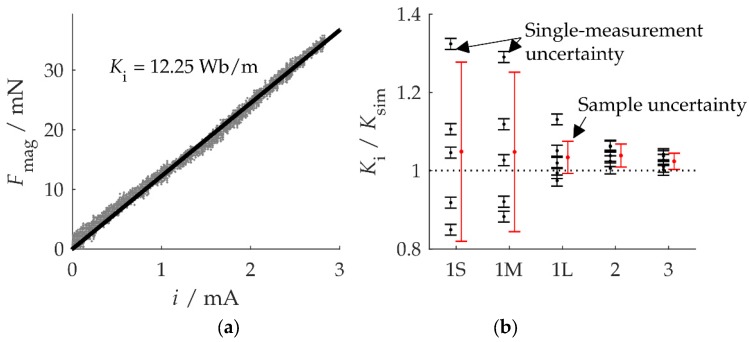
Identification of the coupling coefficient by way of a magnetic-force measurement: (**a**) Magnetic force for setup 2 as a function of the coil current and identified coupling coefficient *K*_i_; (**b**) results for *K*_i_ in five subsequent measurements for every setup. The sample uncertainty (red error bar) is much bigger for small clamping lengths, which indicates effects not included in the single-measurement uncertainty Equation (10).

Figure 6 shows the measured magnetic force $F_{\mathrm{mag}}$ as a function of the coil current i for setup 2. The slope of the linear fit is the coupling coefficient $K_{i}$ from Equation (10). The measurement results are in excellent agreement with Equation (10). They also indicate a linear elastic behavior, or else the slope of the measured curve would not be constant.

However, the measurement is more difficult for shorter clamping lengths, as Figure 6b shows for five single measurements for each coil setup. The black dots with error bars $u_{K_{i}}$, computed by Equation (10), mark single measurements, while the red error bars mark the overall uncertainty of the result of five subsequent measurements according to $u_{K_{i,\Sigma }}=s_{K_{i}}\cdot t_{\alpha ,n-1}/\sqrt{n}$. Here, $s_{K_{i}}$ is the sample standard deviation, n=5 is the number of measurements, and $t_{\alpha ,n-1}$ denotes the quantile of Student’s probability distribution function with n−1 degrees of freedom at a confidence level of 1−α=95% [23]. The scatter in the repeated measurements is much stronger than $u_{K_{i}}$ would suggest, so there must be additional influences.

In fact, the large sample standard deviation is a consequence of the sensitivity of the deflection measurement to laser triangulation. The deflection of a cantilever element depends on the position of the element along the cantilever. Inadvertently focusing the laser beam on the wrong spot results in a deflection value error, which will translate to a coupling coefficient error. The same may be said about an inadvertently oblique (instead of normal) incidence of the laser beam on the cantilever surface. In our series of measurements, we removed and then re-installed the triangulation sensor before each measurement to get an idea of the magnitude of these effects. It is obvious from Figure 6b that the error increases with decreasing clamping lengths. It follows that Equation (10) substantially underestimates the error of the identified coupling coefficient at small clamping lengths.

### 4.3. Comparison and Discussion

**Figure 7:**
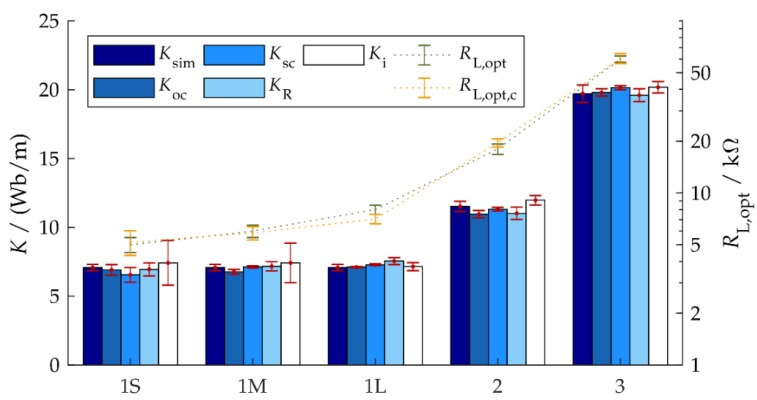
Comparison of the simulated and measured coupling coefficients of five harvester implementations and the measured and predicted optimum load resistance.

The results of determining the electromagnetic coupling coefficient K by different methods are shown in Figure 7 and Table 3. For $K_{i}$, the sample mean of five measurements was used at a confidence level of 95%, as explained in Section 4.2.

On average, the experimentally determined coupling coefficients deviate by about 3% from the simulated value $K_{\mathrm{sim}}$. This clearly shows the appropriateness of all approaches. The maximum observed deviation was between 5% and 8%.

As to the uncertainties of the experimentally identified coupling coefficients, $K_{\mathrm{oc}}$ and $K_{\mathrm{sc}}$ showed the best performance with a relative uncertainty of 3% (average for all setups), followed by $K_{R}$ with about 4%. The large errors in $K_{i}$ for short clamping lengths were already discussed in Section 4.2.

As the different coupling-coefficient measurement methods agree well, one can select one’s preferential method using other criteria. The open circuit method requires only one resonance curve to provide the coupling coefficient K, the mechanical damping factor $\zeta_{m}$, and the resonance frequency $f_{r}$, which can be equated to the natural resonance frequency $f_{n}$ for small $\zeta_{m}$. Hence, the analysis of a harvester only takes a short time. This can be used to predict the optimum load resistance depending on the vibration strength. The mechanical damping increases with stronger vibration amplitudes [24,25], and therefore the optimum load decreases with the amplitude by Equation (5). Determining the optimum load for the full range of vibration amplitudes would take much longer than the open circuit method. Knowing the mechanical damping coefficient for different vibrations amplitudes, one can predict $R_{L,\mathrm{opt}}$.

The short circuit method is perfect in theory but less suitable in practice because it requires knowledge of the mechanical damping coefficient and because the high damping under short circuit conditions is associated with a damped oscillation, which is not easy to measure.

The constant current method produces precise results and is easy to implement, but the calculation of the force from the cantilever beam deflection is error-prone, as discussed in Section 4.2. A direct force measurement would be preferable from this point of view, but, of course, is also more complex to implement.

As a result of the good agreement of the coupling coefficients estimated by various measurement methods or simulations, the prediction of the optimum load resistance by Equation (5) also agrees well with the measured optimum load resistance $R_{L,\mathrm{opt}}$, as can be seen in Table 2. The average and maximum deviations were 5% and 14%, respectively, in our experiments.

## 5. Summary

An important design parameter of electromagnetic vibration energy harvesters is their electromagnetic coupling coefficient, which influences the output power. There are various ways of experimentally identifying the coefficient, but the agreement of the respective methods and their advantages or disadvantages have not been considered until now in the open literature. In this work, we have experimentally tested four methods for the direct or indirect measurement of the coupling coefficient and have compared the results with data from FE simulations. The extracted coupling coefficients agree to within 3% among all methods tested. This validates both the measurement methods and the theory of electromagnetic coupling and optimum load resistance. This means that the coupling behavior can be predicted on the basis of simulation data alone and the optimum load resistance can be predicted on the basis of estimated (measured) mechanical damping coefficients. In this situation, the method of choice is the one that involves the least measurement effort. This is the open circuit method, which provides all necessary parameters within a short measurement time.

## Figures and Tables

**Table 1:** Parameters of the coils used in different harvester implementations.

Setup No.	Description	*ℓ*/mm	*f*_r_/Hz	*N*	*D*_w_/µm	*R*_C_/Ω	*â*/m/s^2^
1L	Coil 1, long	27	51.2	1300	50	226	0.75
1M	Coil 1, medium	22	65.3	1300	50	226	1
1S	Coil 1, short	18	81.2	1300	50	226	1
2	Coil 2	27	51.2	2115	40	880	0.75
3	Coil 3	27	51.2	3620	30	1707	0.75

**Table 2:** Measured and calculated optimum load resistances and measured maximum load power at an acceleration amplitude of *â* = 0.75 m/s^2^. Setups 1M and 1S were characterized at higher amplitudes (*â* = 1 m/s²), but the results were scaled to *â* = 0.75 m/s^2^.

Setup No.	*R*_C_/kΩ	*R*_L,opt_/kΩ	*R*_L,opt,c_/kΩ	*P*_max_/µW
1L	0.23	8	7.1	257
1M	0.23	6	5.9	179
1S	0.23	5	5.2	94
2	0.88	18	19.6	223
3	1.7	60	60.6	253

**Table 3:** Simulated and measured coupling coefficients. The data are visualized in Figure 7.

Setup No.	Coupling Coefficient in Wb/m				
	*K*_sim_	*K*_oc_	*K*_sc_	*K*_R_	*K*_i_
1L	7.1	7.1	7.3	7.6	7.2
1M	7.1	6.8	7.1	7.2	7.4
1S	7.1	6.9	6.6	7.0	7.4
2	11.5	11.0	11.3	11.0	12.0
3	19.7	19.8	20.2	19.6	20.2

**Figure A1:**
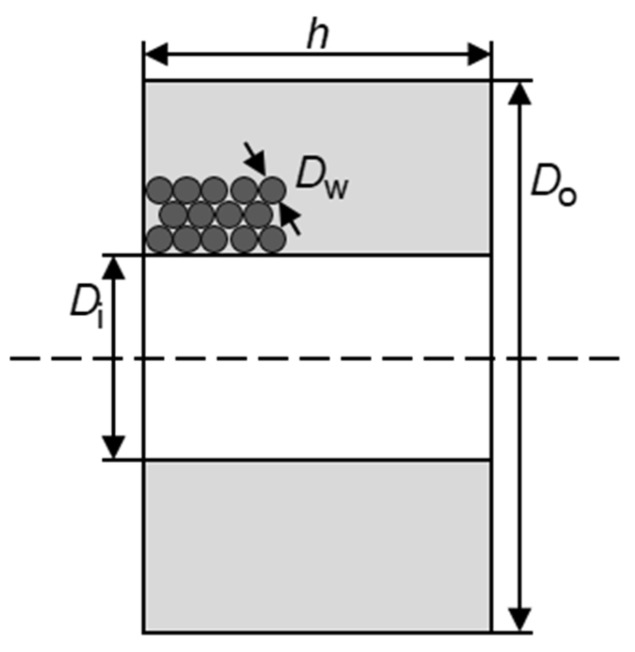
Cross-section of an air coil. The grey area is filled with windings (some wire cross-sections are shown as examples).

## Supplementary Materials

The following are available online at https://www.mdpi.com/2072-666X/10/12/826/s1.

## Appendix A

An air coil (see Figure A1) consisting of a wire with a total length of $\ell_{\mathrm{tot}}$, a resistivity of $\rho_{\mathrm{el}}$, and a constant cross-sectional area of $A_{\mathrm{wire}}$ has the resistance

(A1) $$R_{C}=\rho_{\mathrm{el}}\frac{\ell_{\mathrm{tot}}}{A_{\mathrm{wire}}}.$$

The total length is calculated by the number of coil turns N and the mean length of one coil turn $\bar{\ell }$. With a wire diameter much smaller than the outer coil dimensions, and therefore with many coil turns, the total length is

(A2) $$\ell_{\mathrm{tot}}=N\bar{\ell }=N\pi (D_{o}+D_{i})/2.$$

As measured by the outer coil dimensions, a single winding encloses the area

(A3) $$A_{\mathrm{winding}}=\frac{h(D_{o}-D_{i})}{2N}.$$

The ratio between the winding area and the wire cross-sectional area is expressed by the filling factor $F:=A_{\mathrm{wire}}/A_{\mathrm{winding}}$. One always has F<1 because of imperfect wiring and the wire coating. Applying these equations to Equation (19), we arrive at the following expression for the coil resistance as a function of the coil turn number N:

(A4) $$R_{C}=\rho_{\mathrm{el}}\frac{\pi N^{2}(D_{o}+D_{i})}{F(D_{o}-D_{i})h}$$
